# Is this a “lucky LEG”? A retrospective analysis of the management of Lower-Entity Gustilo open fractures requiring flap coverage in the lower extremity

**DOI:** 10.1016/j.jpra.2026.01.027

**Published:** 2026-01-23

**Authors:** Joachim N. Meuli, Julien Gisiger, Daniel Wagner, Pietro G. di Summa

**Affiliations:** a Department of Plastic and Hand Surgery, Lausanne University Hospital, University of Lausanne, Rue du Bugnon 46, Lausanne, Vaud, 1011, Switzerland; b Department of Orthopedics and Traumatology, Lausanne University Hospital, University of Lausanne, Rue du Bugnon 46, Lausanne, Vaud, 1011, Switzerland

**Keywords:** Open fractures, Flap coverage, Bone infection, Bone union

## Abstract

**Background:**

Most of the literature about soft-tissue coverage of open fractures is focused on Gustilo-Anderson IIIB/IIIC. A significant proportion of the flaps performed for coverage after open fractures is however performed for Gustilo-Anderson types I to IIIA which develop soft-tissue damage over days after injury. This study aims at analyzing this specific subset of open fractures regarding characteristics, management and outcomes.

**Methods:**

A retrospective study was conducted on patients who benefited from a pedicled or free flap coverage for open fractures of the lower extremity. Bone union and rate of deep infection were compared between patients presenting open fractures type I to IIIA (Lower Entity Gustilo—LEG) with secondary soft-tissue damage and patients who presented open fractures type IIIB and IIIC (Higher Entity Gustilo) with immediate soft-tissue damage.

**Results:**

Eighteen patients were included, seven of which were in the first group (LEG) and 11 in the second group (HEG). Time from injury to coverage was significantly longer for the LEG group (23.6 *Vs* 9.5 days) and so was the delay between definitive fixation and coverage (6.6 *Vs* 0.0 days). Rate of bone union was lower and rate of deep infection was higher in the HEG group (64% *Vs* 0%, *p* = 0.019).

**Conclusion:**

Open fractures of the lower extremities type Gustilo-Anderson I to IIIA (LEG) which develop soft-tissue damage after injury eventually requiring flap coverage seem to have a lower risk of deep infection and delayed union despite longer delay between trauma and coverage as well as between definitive fixation and coverage.

## Introduction

Open fractures are characterized by an open wound in the skin at the site of the broken bone. Since the skin barrier has been lost, the potential for contamination is high, increasing the risk of complications such as septic non-union. These complications, in turn, results in high economic costs and associated morbidity.^1^ To predict the risk of contamination, the most popular classification is the one Gustilo and Anderson presented in 1976^2^ and refined in 1984.^3^ Studies have shown an actually fairly limited reproducibility of this classification^4^^,^^5^ but it remains the most commonly used worldwide. From a reconstructive surgery perspective, the main issue is that this grading system evaluates soft-tissue damage only at the time of presentation (as in the original study) or at the latest at the time of first debridement (according to more recent guidelines^6^). Soft tissue damage can however also be progressive and sometimes appears over time after injury, especially in compression trauma or in frail patients. As such, open factures initially classified as Gustilo-Anderson type I to IIIA might present with more extensive soft-tissues damages in the days following trauma. If these defects appear before the definitive fixation, the management of these fractures becomes very similar, in terms of soft-tissue coverage, to Gustilo-Anderson types IIIB or IIIC. However, elapsed time from injury to fixation or from injury to final coverage may be potentially longer in such patients, with consequences on healing time and infection control. Interestingly, given the much higher incidence of type I to IIIA compared to type IIB and IIIC, a significant proportion of the flap-based coverage procedures for open fractures is performed on these subtypes while most of the available literature focuses on type IIIB-IIIC.

The goal of this study was to compare lower entity Gustilo-Anderson (LEG) open fractures (type I-IIIA) which presented secondary soft-tissue damage and required a flap coverage, with higher entity Gustilo-Anderson (HEG) open fractures (type IIIB-IIIC). We hypothesize that lower grade fractures requiring a flap coverage because of secondary soft-tissue damage present a similar management but different risk profile than high grade fractures.

## Materials and methods

### Study design

This exploratory study was designed as a monocentric retrospective study at Lausanne University Hospital, based on a prospectively maintained database including all flaps performed for lower limb open fractures. All adults (>18 years old) patients who presented with an open fracture of the lower limb and who benefited from a flap-based coverage surgical procedure (either pedicled flap or free flap) because of a soft-tissue defect between January 2019 and April 2022 were included. This limited timeframe was selected because of a change in Gustilo-Anderson grading in our institution. Until 2019, grading was performed by orthopedic surgeons while cases after this date were assessed by a combined orthoplastic team. Patients were followed from the time of initial presentation until complete healing of soft tissues and bones (radiological and clinical bone union). Patients who presented minimal soft-tissue defects initially but developed, before the definitive fixation, soft tissue damage extensive enough to require a flap were grouped in the first arm of our study (Lower Entity Gustilo-Anderson—LEG). Patients who presented soft-tissue defects large enough to require a flap coverage at the time of injury or after the first debridement were grouped in the second arm (High Entity Gustilo-Anderson—HEG). Patients in which a flap was performed to cover a defect in another location than the open fracture, patients with incomplete data regarding trauma, patients lost to follow-up and patients with follow-up shorter than 12 months were excluded. Patients with open fractures Gustilo-Anderson I to IIIA treated by primary closure or skin grafting at the time of definitive fixation and who required a flap coverage at a later stage because of soft-tissue deficiency caused by chronic infection were excluded as well because the orthoplastic management of these cases is fundamentally different from acute soft-tissue injury. In the absence of previous literature on this topic, sample size was determined by the total number of patients treated in the above-mentioned period and considered adequate given the exploratory nature of this study. Population’s characteristics, intervention details, and outcomes were collected from the hospital electronic medical records. Reporting in this manuscript adheres to the STROBE guidelines.

### Surgical strategy

The initial phase of management involved excision of the wound and debridement, alongside fracture reduction and stabilization by the orthopedic trauma team. Initial debridement was planned within 24 h of admission, irrespective of the case being a primary admittance or a secondary transfer from another trauma facility. Gustilo-Anderson grading was performed by a multidisciplinary orthoplastic team. Once the injury site was deemed cleaned enough and soft-tissue damage had stabilized, definitive bone stabilization using intramedullary nails, plates, external fixators or a combination of these techniques was performed. Soft tissue reconstruction was performed by the plastic surgery team with free flaps or pedicled flaps and took place ideally at the same time as definitive stabilization. The number of dressing changes before soft-tissue coverage was minimized as possible and these were systematically performed using negative pressure wound therapy (NPWT) dressings in the operating theatre.

### Investigated variables and outcomes

The following patients characteristics were gathered: age and gender, comorbidities (tobacco use, renal failure, high blood pressure, arteriopathy and diabetes), Gustilo-Anderson classification, localization of the injury, history of previous surgery at the same site, presence of associated injuries, referral from another hospital, delay between injury and the first debridement, time and type of initial stabilization, time and type of definitive fixation, number of debridements before definitive fixation and coverage as well as time and type of coverage. Procedures performed in other institutions and previous hospitalizations prior to admission at the Lausanne University Hospital were not considered because this data could not be assessed accurately.

Primary bone union was defined as the main outcome and defined as bone healing achieved within 6 months of the initial definitive fixation without secondary procedures. If secondary procedures were necessary to achieve bone healing between 6 and 24 months, it was defined as delayed bone union. If no bone healing was achieved by 24 months or by the time of the latest recorded appointment, it was defined as non-union.

Secondary outcomes of interest were the occurrence of deep infection, the time to complete bone union as well as the time to full weight bearing. Because there is no standardized definition of bone infection in the setting of open fractures, we used the US Centers for Disease Control and Prevention (CDC) definition of deep incisional surgical site infection as surrogate. This definition has been used in recent large monocentric and multicentric studies.^7^^,^^8^ It is worth noting that this definition requires the identification of micro-organisms in deep soft tissue but not in bone and only if purulent drainage is absent. Because of this, we gathered supplementary data regarding the results of routine bone sampling performed at the time of definitive fixation and in cases of deep incisional surgical site infection (see discussion section). The grown organisms were recorded in all cases. Time to bone union was calculated from the date of the injury to the consolidation of the bone (radiological bone union). Time to full weight bearing was calculated from the date of injury to the date the patient was allowed and able to bear full weight on the injured limb. Patient with missing data regarding the primary outcome were excluded. If missing data concerned a secondary outcome, patients were excluded from the specific analysis of this outcome.

### Statistical analysis

Continuous variables are presented as mean (SD) while discrete data are presented as full numbers or percentage. Differences in characteristics and outcomes were assessed using two‐sided exact Fisher’s test for categorical variables and ANOVA for continuous variables. All analyses were performed using the R Project for Statistical Computing version 4.4.1. A *p*-value < 0.05 was considered as statistically significant.

## Results

### Patients characteristics

**Figure 1:**
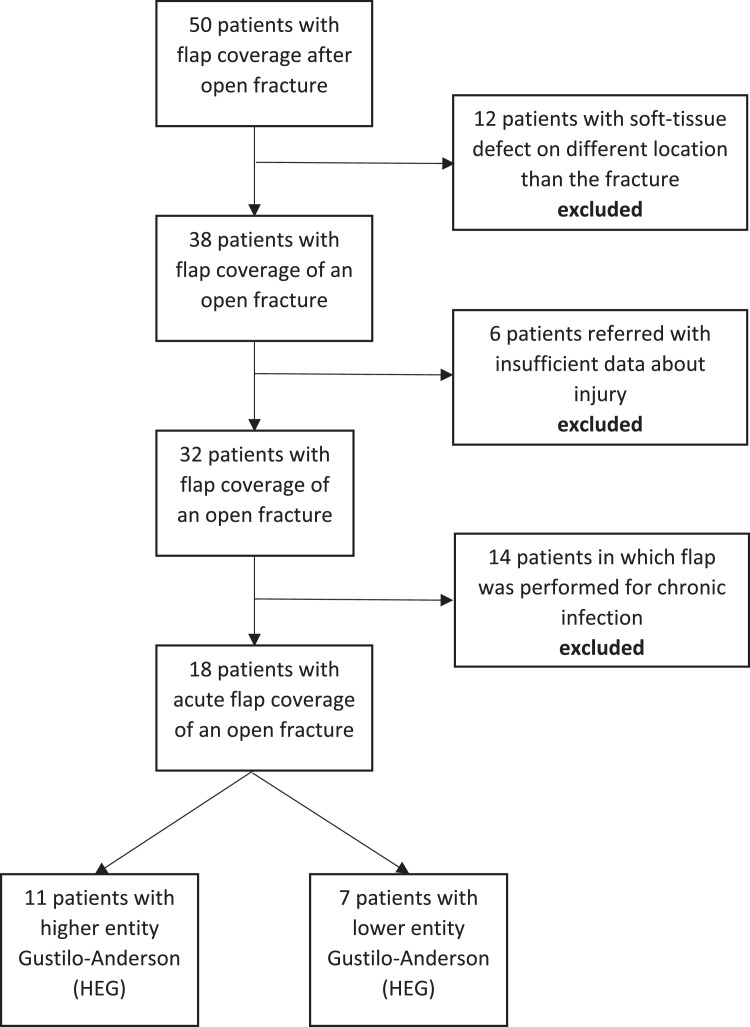
Flowchart.

The study population consisted of 50 patients. Twelve patients presented different sites of fracture and soft-tissue defect, six patients were referral cases with insufficient data and 14 patients were chronic cases with soft-tissue damage appearing long after definitive fixation and primary closure. All these patients were excluded, leaving 18 patients included who all completed follow-up and analysis (Figure 1: flowchart). Seven of these were included in the first group (Lower Entity Gustilo with secondary need of soft tissue coverage—LEG) and 11 in the second group (Higher Entity Gustilo with immediate need of soft-tissue coverage- HEG). Patients’ and injury characteristics for each arm are presented in Table 1. There was no difference between both groups except for Gustilo-Anderson type, injury mechanism and fracture localization. As expected, the LEG group consisted of type I, II and IIIA exclusively while the HEG group consisted of type IIIB and IIIC exclusively. In the LEG group, the open fractures were predominantly located at the distal tibia (86%) whereas they were distributed over the entire lower extremity in the HEG group. Falls were the predominant mechanism in the LEG group while motor vehicle accidents were the leading cause in the HEG group.

**Table 1:** Characteristics of participants and injury; mean (SD) and *N* (%).

	Total	Immediate	Secondary	*p*-value
	*N* = 18	*N* = 11	*N* = 7	
Age	47.2 (16.9)	50.5 (13.8)	42.1 (21.0)	0.324
Male (%)	16 (88.9)	10 (90.9)	7 (87.5)	1.000
BMI	24.3 (3.4)	23.8 (3.2)	25.1 (3.7)	0.432
Hypertension (%)	2 (11.1)	1 (9.1)	1 (14.3)	1.000
Smokers (%)	7 (38.9)	4 (36.4)	3 (42.9)	1.000
Renal failure (%)	1 (5.6)	1 (9.1)	0 (0.0)	1.000
Diabetes (%)	1 (5.6)	1 (9.1)	0 (0.0)	1.000
Arteriopathy (%)	1 (5.6)	1 (9.1)	0 (0.0)	1.000
Previous surgery at same site (%)	6 (33.3)	3 (27.3)	3 (42.9)	0.864
Injury mechanism				**0.044**
Motor vehicle accident (%)	7 (38.9)	6 (54.5)	1 (14.3)	
Fall (%)	8 (44.4)	2 (18.2)	6 (85.7)	
Work accident (%)	2 (11.1)	2 (18.2)	0 (0.0)	
Crush injury (%)	1 (5.6)	1 (9.1)	0 (0.0)	
Fracture localization				**0.020**
Patella (%)	1 (5.6)	0 (0.0)	1 (14.3)	
Proximal tibia (%)	5 (27.8)	5 (45.5)	0 (0.0)	
Intermediary tibia (%)	3 (16.7)	3 (27.3)	0 (0.0)	
Distal tibia (%)	8 (44.4)	2 (18.2)	6 (85.7)	
Foot (%)	1 (5.6)	1 (9.1)	0 (0.0)	
Associated injuries (%)	12 (61.1)	6 (54.5)	5 (71.4)	0.826
Gustilo-Anderson classification (%)				**0.001**
Type I	1 (5.6)	0 (0.0)	1 (14.3)	
Type II	4 (22.2)	0 (0.0)	4 (57.1)	
Type IIIA	2 (11.1))	0 (0.0)	2 (28.6)	
Type IIIB	8 (44.4)	8 (72.7)	0 (0.0)	
Type IIIC	3 (16.7)	3 (27.3)	0 (0.0)	
Referral from other hospital	10 (55.6)	6 (54.5)	4 (57.1)	1.000
Follow-up	30.0 (14.0)	34.0 (14.0)	25.0 (11.0)	0.170

Values highlighted in bold are values <0.05.

### Management

There was a difference in the time from injury to initial fracture stabilization between the two groups (Table 2). Patients in the HEG group presented no delay from injury to initial stabilization while the patients in the LEG group had an average delay of 0.4 days (9.6 h). The time from injury to definitive fixation was longer in the LEG group, in consistence with the progressive appearance of soft tissue damage (16.9 days *Vs* 9.5 days, *p* = 0.059). Patients in the HEG group underwent 2.0 debridements on average while patients in the LEG group underwent 2.6 debridements. This difference was however not significant (*p* = 0.512) 27% of the soft-tissue defects in the HEG group were covered with free flaps while this was the case for 86% of the defects in the LEG group (*p* = 0.053). The time from injury to coverage was significantly longer for the LEG group (23.6 *Vs* 9.5 days, *p* = 0.004) and so was the delay between definitive fixation and coverage (6.6 *Vs* 0.0 days, *p* = 0.046). This difference was caused by three of the seven patients in the LEG group who did not benefit from a simultaneous soft tissue coverage and definitive fixation but were instead covered at a later stage. One was delayed because of medical complications (drug induced agranulocytosis), the second one was planned with a delay because a chimeric medial femoral condyle and fascio-cutaneous flap was necessary and the delay in the third case was caused by a transfer from another institution. There was no difference in techniques either for initial fixation or for definitive fixation.

**Table 2:** Treatment characteristics; mean (SD) and *N* (%).

	Total	Immediate	Secondary	*p*-value
	*N* = 18	*N* = 11	*N* = 7	
Time from injury to initial fixation (days)	0.2 (0.4)	0.0 (0.0)	0.4 (0.5)	**0.016**
Initial fixation technique				1.000
External fixation (%)	16 (88.9)	10 (90.9)	6 (85.7)	
K-wires/osteosuture (%)	1 (5.6)	1 (9.1)	1 (14.3)	
Time from injury to definitive fixation (days)	12. 4 (8.2)	9.5 (4.4)	16.9 (10.9)	0.059
Number of debridements	2.2 (1.7)	2.0 (1.6)	2.6 (2.1)	0.512
Definitive fixation technique				0.221
Plates and/or screws (%)	13 (72.2)	7 (63.6)	6 (85.7)	
Intramedullary nail (%)	3 (16.7)	3 (27.3)	0 (0.0)	
External fixation (%)	1 (5.6)	1 (9.1)	0 (0.0)	
Osteosuture (%)	1 (5.6)	0 (0.0)	1 (14.3)	
Time from injury to coverage (days)	14.9 (11.0)	9.5 (4.4)	23.6 (12.9)	**0.004**
Coverage technique				0.053
Free flap (%)	9 (50.0)	3 (27.3)	6 (85.7)	
Pedicled flap (%)	9 (50.0)	8 (72.7)	1 (14.3)	
Time from def. fixation to coverage (days)	2.6 (6.9)	0.0 (0.0)	6.6 (10.2)	**0.046**

### Outcomes

**Figure 2:**
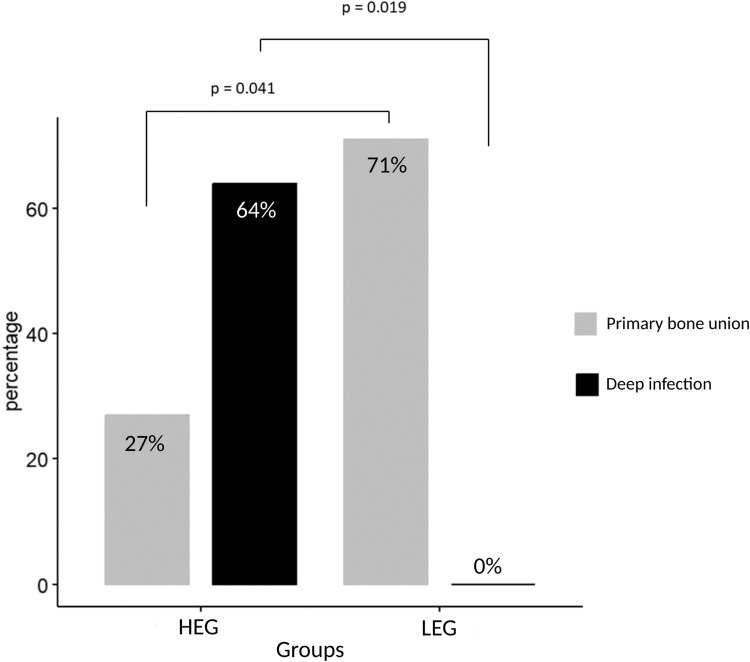
Occurrence of osteomyelitis and bone union in LEG group and HEG group.

Primary bone union was achieved in 71% of patients in the LEG group with secondary soft-tissue damage, while this was achieved in only 27% of patients in the HEG group (*p* = 0.041). Including delayed bone union, bone healing was eventually achieved in 86%, respectively 82% of the patients at an average follow-up of 30 months. Time to bone union was shorter in the LEG group, without statistical significance (7.3 months for LEG group *Vs* 16.7 months for HEG group, *p* = 0.06). Deep infection occurred in seven out of 11 patients (64%) in the HEG group, compared to none of the seven patients (0%) in the LEG group (*p* = 0.019). There was no difference in time to full weight bearing. Details are presented in Fig. 2 and Table 3.

**Table 3:** Outcomes.

	Total	Immediate	Secondary	*p*-value
	*N* = 18	*N* = 11	*N* = 7	
Deep surgical site infection (%)	7 (38.9)	7 (63.6)	0 (0.0)	**0.028**
Primary bone union	8 (44.4)	3 (27.3)	5 (71.4)	**0.041**
Definitive bone union	15 (83.3)	9 (81.8)	6 (85.7)	0.240
Time to bone union (months)	12.9 (9.6)	16.7 (10.3)	7.3 (5.2)	0.060
Time to full weight bearing (months)	7.2 (5.0)	6.9 (4.6)	7.6 (5.9)	0.770

## Discussion

In the setting of open fractures included in this exploratory study, the rate of primary bone union was significantly higher and the rate of deep infection significantly lower for patients developing secondary soft-tissue defects compared to patients with immediate soft-tissue defects. Overall, these results seem to confirm that the infection rate correlates with the severity of the soft-tissue traumatism present at the time of injury. Indeed, secondary soft-tissue damage due to the progression of skin ischemia does not seem to carry the same risk of infection, even when it results in secondary debridement and defects as extensive as immediate damage. This tends to confirm the high predictive value of the Gustilo-Anderson classification regarding infection rates (as all immediate soft-tissue damage fractures were, by definition, type IIIB and IIIC and all secondary soft-tissue damage fractures were type I to IIIA). This however puts into question whether the recommendations from published literature regarding the time from injury to debridement, from fixation to coverage and from injury to coverage apply to Lower Entity Gustilo fractures (I-IIIA). The initial recommendation of a maximum delay of 24 h from injury to debridement for all open fractures is found in both the guidelines for management of orthopedic trauma of the American College of Surgeons and in the National Clinical Guideline Center in the United Kingdom. This is largely based on the work of Hull et al. who found a linear increase in the rate of infection for each hour of delay.^9^ This increase was more important for higher than for lower Gustilo-Anderson types but no specific analysis was performed for the subset of lower entity open fractures with secondary soft-tissue damage. Given that the soft-tissue injury appears over several days in this subgroup, sequential debridement and longer delay seems inevitable. An interesting observation of our study was the predominance of distal leg location for open fractures with secondary soft-tissue damage, reflecting the low tolerance of this area to post-traumatic oedema and the general lower tolerance of the distal leg to trauma and ischemia. The second recommendation regarding the time between definitive fixation and coverage was essentially developed by Gopal et al.^10^^,^^11^ who advocated for simultaneous fixation and soft tissue coverage with later studies confirming these results^8^^,^^7^^,^^12^ and highlighting the predominant role of this “fix and flap” approach relative to other timing recommendations.^13^ The third recommendation regarding the timing between injury and coverage was initially investigated by Godina et al.^14^ who insisted on early soft-tissue coverage within 72 h of trauma, but more recent studies reported an acceptable threshold up to 7 days.^15^, ^16^, ^17^, ^18^ The LEG group of our study was managed entirely outside of these last two recommendations with an average delay between definitive fixation and coverage of 6.6 days and an average time between trauma and coverage of 23.6 days. We nonetheless observed no case of deep infection in this group of “lucky” LEG. This observation, combined with the high bone union rate, makes us wonder if the management of this specific subset of fractures could be delayed if needed without significantly increasing the infection risk. One peculiar result we observed was the absence of any difference in time to full weight bearing between both groups despite higher primary bone union rate in the LEG group. This might be due to imperfect correlation between radiological assessment and function or due to the increased use of intramedullary nailing in the HEG group allowing almost immediate full weight bearing, before complete bone union.

The 64% deep infection rate we report in the HEG group might seem unusually high. Comparatively, Gustilo et al.^3^ reported a 48% deep infection rate in 1984 across type IIIB and IIIC fractures for example but their criteria for infection were not clearly defined. Moreover, 95% of the open fractures in this seminal paper were covered with split-thickness skin grafts only, suggesting that the soft-tissue injuries were significantly different from those included in our study. In 2000, Gopal et al^10^ reported a 30% deep bony infection rate in patients with type IIIB/IIIC open fractures and coverage >72 h after trauma. The criteria for infection as well as the timing of definitive fixation were unfortunately not reported. Around the same time, Hertel et al^19^ analyzed a population more similar to our HEG group, namely patients with open fractures type IIIB and IIIC who required coverage with free or pedicled muscle flaps and reported a 14% overall osteomyelitis rate, the later being defined as clinical signs of infection combined with positive bone cultures. In their cohort of 29 patients, 14 underwent definitive fixation and coverage on the day of injury and none of these developed an infection. The remaining 15 patients benefited from definitive fixation and coverage on average 4.4 days after trauma and in this subset, the osteomyelitis rate was 27%. Olesen et al^18^ also followed a study design similar to ours focused on flap coverage and reported a much higher infection rate at 48%. Overall, studies reported infections rates ranging from 0% to 55% depending on the timing of fixation and/or of coverage.^20^, ^21^, ^22^, ^23^ This extremely wide range is likely the consequence of varying definitions of “infection” across papers with some authors reporting on clinical surgical site infection and others reporting on microbiologically documented bone infection or on combination of both. On top of this, the reproducibility of the Gustilo-Anderson classification is notoriously low and it is likely that the fractures included in these studies were actually associated with various degrees of traumatic energy and soft-tissue injury, therefore with varying risks of infection.

The most plausible explanation for the deep infection rate we observed lies in a combination of patient selection bias and excessive delay in management. Because we included only patients who benefited from a pedicled or free flap-based coverage, we excluded the patients found in other studies with Gustilo-Anderson IIIB/IIIC fractures who are covered with skin-grafts. These patients are likely to have suffered lighter soft-tissue damage and are potentially at lower risk of infection. The 9 days average delay between trauma and definitive fixation combined with coverage we observed in the HEG group likely contributed as well. As mentioned previously, there is indeed ample evidence in the literature supporting early coverage for Gustilo-Anderson type IIIB and IIIC open fractures and even if authors do not agree on the exact timing, 9 days certainly falls within the “intermediate” or “delayed” group of most studies for which the infection risk is significantly increased. The previously mentioned study from Olesen et al. found a 60% infection rate in flap-covered open fractures in which the coverage took place >7 days after injury. We analyzed our cohort for a similar pattern but could not reproduce this difference possibly because of the limited number of patients included.

We do not believe that anti-infectious therapy played a role in the results of this study. Based on studies which showed that deep infections were caused mostly by nosocomial organisms resistant to the initial antibiotic prophylaxis aimed at environmental flora,^24^ the British Standard for Management of Open Fractures^6^ recommends a dual phase antibiotic prophylaxis. Standard practice in our center extends beyond this as bone samples are routinely taken at the time of definitive fixation followed by tailored anti-infectious therapy if germs are found, in a fashion similar to Aljawadi et al.^21^ This practice is debated and the previously mentioned guidelines are rather unspecific regarding this matter, advocating against “wound cultures at time of wound excision” as these do not correlate with later infection. The two studies supporting this recommendation^25^^,^^26^ however reported on wounds swabs only and not on bone samples, leaving the debate open. Aljawadi et al. showed no correlation between bone union rate and positive growth from bone samples taken at the time of definitive fixation. We performed the same analysis in our study with similar results. Bone sampling at the time of definitive fixation was recorded for 13 (72%) patients, with six positive bone cultures and seven negative ones. Sampling was missing for the remaining five patients. Out of the six patients with positive bone culture, three developed clinical osteomyelitis but the pathogens involved did not match the pathogens found at definitive fixation. A study tailored to address this specific question would be very useful to determine if this practice has value.

### Limitations

This study has several limitations. The most obvious is its exploratory nature on a topic that has not yet investigated in depth, with limited sample size and a retrospective single center design. Retrospective analyses can introduce bias as the data collection is not specifically tailored to address the study question. The definition of bone infection used might differ from some other studies and therefore impact infection rates. There is however no standardized and widely accepted such definition in the setting of open fractures despite critical need. Our results only show an association, and no causality can be demonstrated. We were limited to a single medical center and our results as susceptible to bias induced by local protocols, customs, and constraints. The trends observed cannot be directly generalized to other settings and healthcare systems. Personal bias has been rendered less likely by the fact that several contributors performed data collection and data analysis, but it cannot be fully excluded. Overall, we urge readers not to change any current practice patterns based on this early exploratory study but encourage further data collection and reproduction of similar studies to confirm or infirm the trends we observed.

## Conclusion

Open fractures of the lower extremities type Gustilo-Anderson I to IIIA (lower entity Gustilo, LEG) which develop soft-tissue damage after injury and require flap coverage seem to have a significantly higher rate of bone union and lower risk of deep surgical site infection than open fractures Gustilo-Anderson IIIB and IIIC, despite longer delay between trauma and coverage as well as between definitive fixation and coverage.

## Funding sources

The authors did not receive any funding for this study.

## Ethical approval

Ethical approval for this study was obtained from the local ethical committee (number 2023-00081).

## Declaration of competing interest

Pietro G. Di Summa is a Deputy Editor for JPRAS and was not involved in the editorial review or the decision to publish this article. All remaining authors declare no conflict of interest.

## Appendix Supplementary materials

Image, application 1
